# Incidence of urinary incontinence after hip fracture surgery and associated risk factors: a prospective study

**DOI:** 10.1186/s12877-023-04597-4

**Published:** 2024-01-02

**Authors:** Marta Arroyo-Huidobro, Josefa López de la Fuente, Mar Riera Pagespetit, Oscar Macho Perez, Jaume Roig Morera, Anna Maria Abelleira López, David Aivar Casanova, Esther Garcia-Lerma, Carlos Pérez-López, Alejandro Rodríguez-Molinero

**Affiliations:** 1 Hospital Residencia Sant Camil, Consorci Santiari Alt’Pènedes i Garraf, Sant Pere de Ribes, Barcelona, Spain; 2 Geriatrics Unit, Department of Internal Medicina, Hospital Clinic de Barcelona, C. de Villarroel, 170, Barcelona, 08036 Spain; 3 Geriatrics Area, Hospital Vilafranca, Consorci Santiari Alt’Pènedes i Garraf, Vilafranca del Penedès, Barcelona, Spain; 4 Biostatistics Unit, Bellvitge Biomedical Research Institute (IDIBELL), L’Hospital de Llobregat, Barcelona, Spain; 5 Area de Recerca, Consorci Sanitari Alt’Pènedes I Garraf, Villafranca del Penedès, Barcelona, Spain

**Keywords:** Hip fracture Surgery, Risk factors, Urinary incontinence

## Abstract

**Background:**

The contribution of the postoperative process to developing or worsening urinary incontinence (UI) after hip fracture surgery (HFS) remains unclear. We aimed to evaluate UI incidence and worsening among older patients undergoing HFS, and explore associated risk factors.

**Methods:**

This prospective cohort study included patients ≥ 75 years admitted between October 2019 and October 2021 to the Traumatology Service of three hospitals in the Consorci Sanitari de Alt-Penedès i Garraf (Barcelona, Spain) with hip fracture requiring surgical treatment. UI was assessed using the first two questions of the International Consultation on Incontinence Questionnaire - Short Form (ICIQ-SF) at baseline and at days 30 (± 3 days) and 90 (± 3 days) after HFS. Surgery-related data and post-surgical complications were recorded.

**Results:**

A total of 248 patients with a mean (SD) age of 85.8 (6.78) years were included; 77.8% were female and 154 (62.1%) had UI at baseline. After HFS, 3.24% experienced urinary tract infections (UTIs), 3.64%, acute urinary retention (AUR), 8.57%, constipation, and 53.9%, prolonged catheterization (> 24 h). Fifty-eight patients without baseline UI developed UI at 30 days, resulting in a UI incidence of 61.7% (95% CI 51.1–71.54) between days 0 and 30. Of the 248 patients, 146 (59.1%) experienced worsening of UI. AUR and UTIs were identified as risk factors for UI development and worsening after HFS, respectively.

**Conclusion:**

The incidence of UI in older patients after HFS is significant. Patient management protocols should consider AUR and UTIs to reduce or eliminate the incidence of UI in older patients undergoing HFS.

**Supplementary Information:**

The online version contains supplementary material available at 10.1186/s12877-023-04597-4.

## Introduction

Hip fractures (HFs) are considered a severe health hazard for the aged population and one of the most common geriatric disorders [1]. HF significantly impacts morbidity, with 30‒50% of patients losing their ability to function independently, and mortality, with a 22% mortality rate the first year after HF [1]. Age and the female gender are risk factors for this condition [1, 2]. The prevalence of HF among people aged 50 years and older varies widely across the globe, ranging from more than 500 cases per 100,000 adults (in Denmark) to under 100 cases per 100,000 adults (e.g., South Africa) [3]. In Spain, the National Hip Fracture Registry (*Registro Nacional de Fracturas de Cadera*, or RNFC) reported a total of 10,068 HF cases in 2021, of which 33% corresponded to individuals between 75 and 84 years, 57% to individuals between 85 and 94 years, and 9.2% to individuals over 94 years [4]. Regarding gender differences, the most recent RNFC report showed that 75.4% of registered patients were female [5].

The implant of a full or partial hip prosthesis or osteosynthesis by surgery constitutes the mainstay of treatment [6]. It is often performed within the first 24 to 72 h after HF. The timing of surgery is essential: the earlier the procedure is carried out, the lower the morbidity and mortality rates [7]. HF and the postoperative period are associated with several conditions, such as falls and new fractures, infections, pressure ulcers, delirium, cardiovascular events, and urinary tract infections (UTIs) [8]. Urinary catheterization is a routine procedure before surgery that is often withdrawn within 24 h [9]. Longer catheter use is associated with several urinary problems, including higher rates of UTI and postoperative urinary retention, and may result in hydronephrosis, pyelonephritis, renal insufficiency, bacteriuria, and urinary incontinence (UI) [9, 10].

Despite the existing evidence regarding complications after hip fracture surgery (HFS), it is still unclear how the postoperative process contributes to developing UI or worsening previous UI, and few studies have prospectively assessed UI incidence after HFS [11]. Given the significant medical, economic, and social impact of this condition on older people [12], identifying modifiable risk factors for developing UI after HFS can aid in generating clinical protocols for its prevention. This prospective study aimed to assess the incidence of UI and the evolution of this condition among older patients undergoing HFS and investigate the risk factors associated with UI development and worsening in these patients.

## Methods

### Study design and population

This was a prospective cohort study, including patients admitted to the Traumatology Service of the Consorci Sanitari de Alt-Penedès i Garraf (CSAPG) of the Province of Barcelona who experienced HF due to a fall. CSAPG includes three hospitals: Hospital Sant Antoni Abad (Vilanova i la Geltrú), Hospital Residencia Sant Camil (Sant Pere de Ribes), and Hospital Comarcal de l’Alt Penedès (Vilafranca del Penedès). All patients who were at least 75 years old, had HF that required surgical treatment, and signed an informed consent form were included consecutively between October 1, 2019 and October 1, 2021. Exclusion criteria were the patient’s inability to provide consent and absence of a legal representative or *de facto* guardian, the presence of other associated fractures, the inability to contact the patient and/or primary caregiver by telephone during follow-up after hospital discharge, admission to a hospitalization unit before the HF, and concomitant acute disease at the time of the HF. A baseline interview with HF patients was conducted the same day or the day after their recruitment, between days 0 and 3 after the fracture (i.e., after admission). Researchers followed up patients for 90 days, with two telephone interviews at days 30 (±3 days) and 90 (±3 days).

The study protocol was approved by the Hospital Universitari de Bellvitge independent ethics committee (reference number: PR310/19). This research was conducted following the Helsinki Declaration and the EU General Data Protection Regulation; all personal data were separated from the results (GDPR).

### Objectives and variables

To evaluate the primary objective (incidence of UI), we used the validated Spanish version of the International Consultation on Incontinence Questionnaire - Short Form (ICIQ-SF) [13]. This questionnaire assesses the impact of UI on a 1‒10 scale based on the individual’s perception of: (1) the frequency of urine leakage; (2) the amount of urine loss; and (3) how urine leakage affects the individual’s daily life. Relatives of patients with cognitive impairment completed the fourth question of the ICIQ-SF regarding the situations leading to urine leaks. The answers to the first two questions of the ICIQ-SF (frequency of urine leakage and amount of urine loss) were used to assess UI. Patients with scores other than 0 (i.e., answers other than ‘Never’) in the first two questions were recorded as having UI, whereas those with a score of 0 were considered continent. The ICIQ-SF was administered at baseline and at 30 and 90 days after HFS.

Secondary objectives were UI prevalence and evolution, and the analysis of risk factors associated with developing UI and worsening of UI after HFS. Increased ICIQ-SF scores from baseline were considered a worsening of pre-existing UI in patients with UI at baseline or incident UI.

Variables related to HFS were collected from clinical records, including scores and surgery characteristics, as well as type of anesthesia, from the American Society of Anesthesiologists (ASA) (categories I to VI). Additionally, complications or concomitant processes occurring during hospitalization, such as acute urinary retention (AUR), UTIs (defined as symptomatic UTIs requiring antibiotic treatment), constipation (i.e., those patients requiring enemas were considered as having constipation), time from surgery to ability to sit up (days), catheterization time (hours or days), and reason for catheterization and maintenance after surgery, were collected.

Variables considered at baseline included age, sex, body mass index (BMI) (kg/m^2^), and comorbidities, collected from clinical records and reported by the accompanying caregiver during the baseline interview, as well as university studies, height (m), and weight (kg) reported by the patient during the baseline interview. Patients’ functional and mental status were assessed during the baseline interview using the Barthel scale administered to the patient’s caregiver, the Confusion Assessment Method (CAM), and Pfeiffer’s Short Portable Mental Status Questionnaire (SPMSQ), ranging from 0 to 10, with a threshold for suitability of 5, which were used to evaluate patients’ competency to answer the ICIQ-SF. The Barthel scale scores for the items assessing ambulation, use of stairs, and urination were registered as part of patients’ baseline characteristics.

### Statistical analysis

A sample size of 193 patients admitted for surgical treatment of HF was deemed suitable to estimate an expected cumulative incidence of 25%, with a 95% confidence and a precision of +/- 6% units.

Categorical variables were described as frequencies and percentages, and continuous variables were described as the mean and standard deviation (SD). The incidence of UI (primary objective) was calculated as the incidence proportion on the continent population at baseline (i.e., a score of 0 for questions 1 and 2 of the ICIQ-SF at baseline). UI incidence was assessed during two periods: between day 0 and day 30 after HFS and between day 0 and day 90 after HFS, and was presented as percentage with its 95% confidence interval (CI). The prevalence of UI was calculated at baseline, at 30 days, and at 90 days and presented as percentage with its 95% CI.

Log-binomial models were used to investigate relationships between risk factors of UI and UI incidence and worsening after HFS at 90 days. Variables included in the multivariate models were selected based on their statistical significance in bivariate analyses and clinical relevance. Relative risks, as well as 95% CIs and *p*-values, were calculated. The significance threshold for all analyses was set at a two-sided α < 0.05. Statistical analyses were performed using the R 4.1.0 software.

## Results

### Characteristics of the study population

A total of 331 patients were recruited, of which 53 did not meet eligibility criteria and 30 had missing data, resulting in a study population of 248 patients. Baseline demographic and clinical characteristics of the study cohort are included in Table 1. The mean (SD) age of patients was 85.8 (6.78) years, 77.8% were female, and the mean (SD) BMI was 25.0 (4.4) kg/m^2^. At baseline, 154 (62.1%) patients had UI.

**Table 1:** Baseline demographic and clinical characteristics of study patients, *n* = 248^a^

**Demographic Characteristics**	
Age (years), *n (%)*	
75–79	40 (16.1)
80–84	60 (24.2)
85–89	82 (33.1)
≥90	66 (26.6)
Sex, *n (%)*	
Female	193 (77.8)
Male	55 (22.2)
Height (m) (*n* = 243), *mean (SD)*	1.61 (0.09)
Weight (kg) (*n* = 244), *mean (SD)*	64.5 (12.4)
BMI (kg/m^2^) (*n* = 243), *mean (SD)*	25.0 (4.37)
University studies, *n (%)*	46 (21.5)
**Clinical Characteristics**	
Comorbidities	
Diabetes, *n (%)*	72 (29.0)
Heart disease, *n (%)*	61 (24.9)
Neurovascular disease, *n (%)*	46 (18.5)
Dementia, *n (%)*	69 (27.8)
Parkinson, *n (%)*	11 (4.44)
**Mobility** Ambulation (*n* = 247), *n (%)*	
Independent	130 (52.6)
Independent in wheelchair	11 (4.45)
Need help	91 (36.8)
Dependent	15 (6.07)
Stairs (*n* = 246), *n (%)*	
Independent	109 (44.3)
Need help	61 (24.8)
**Treatment** Drugs before admission (*n* = 248), n *(%)*	
Alpha-blockers	23 (9.27)
Selective alpha2 antagonists	1 (0.40)
Relaxing external sphincter	5 (2.02)
Antidepressants	88 (35.5)
Diuretics	100 (40.3)

BMI, body mass index; SD, standard deviation

^a^Unless otherwise stated

### Surgery variables and Complications

Table 2 summarizes surgery-related data and post-surgery complications. Over half of the study cohort (60.7%) had an ASA III score. Regarding surgical procedures, 59.9% of patients experienced a non-prosthetic surgery and 53.8% of the interventions were intramedullary nailing. The majority of surgical procedures were performed under neuroaxial anesthesia (96.4%). Regarding post-surgery complications, UTIs, AUR, and constipation were experienced by < 10% of patients. Almost half of patients had prolonged catheterization (> 24 h) after HFS.

**Table 2:** Summary of surgery-related data and post-surgical complications

**Surgery and Anesthesia**	
ASA category *n* = 247, *n (%)*	
I	1 (0.40)
II	83 (33.6)
III,	150 (60.7)
IV	5 (2.02)
Unknown	8 (3.24)
Prosthetic surgery *n* = 247, *n (%)*	
Yes	99 (40.1)
No	148 (59.9)
Surgery type *n* = 247, *n (%)*	
Cannulated screws	8 (3.24)
Intramedullary nailing	133 (53.8)
Cemented hemiarthroplasty	26 (10.5)
Uncemented hemiarthroplasty	44 (17.8)
Concrete THA	23 (9.31)
No concrete THA	6 (2.43)
Other	7 (2.83)
Type of anesthesia *n* = 247, *n (%)*	
General	8 (3.24)
Neuroaxial	238 (96.4)
Other region	1 (0.40)
**Post-Surgical Complications**	
Urinary tract infection (*n* = 247), *n (%)*	8 (3.24)
Acute urinary retention (*n* = 247), *n (%)*	9 (3.64)
Constipation (*n* = 245), *n (%)*	21 (8.57)
Time with bladder catheter (*n* = 247), *n (%)*	
< 24 h	114 (46.2)
24–48 h	34 (13.8)
48–72 h	46 (18.6)
3–4 days	22 (8.91)
5–6 days	19 (7.69)
7–8 days	7 (2.83)
≥9 days	5 (2.02)
Prolonged catheterization (> 24 h) (*n* = 248), *n (%)*	133 (53.9)

ASA, American Society of Anesthesiology; THA, Total Hip Arthroplasty

### Incidence and prevalence of urinary incontinence

Of the 94 patients without UI at baseline, 58 developed UI at 30 days, resulting in a UI incidence of 61.7% (95% CI 51.1–71.54) between day 0 and day 30. At 90 days, 49 of the 94 patients without UI at baseline developed UI, resulting in a UI incidence of 52.1% (95% CI 41.57–62.54) between day 0 and day 90. The prevalence of UI at baseline was 62.1% (95% CI 55.74–68.16) and increased at both time points after HFS (Table 3).

**Table 3:** Incidence and prevalence of urinary incontinence

	Baseline (*n* = 248)	30 days (*n* = 248)	90 days (*n* = 228)
Incidence **(***n* = 94*), *n (%)*	NA	58 (61.7)	49 (52.1)
95% CI	NA	51.1–71.54	41.57–62.54
Prevalence, *n (%)*	154 (62.1)	197 (79.4)	175 (76.7)
95% CI	55.74–68.16	73.86–84.29	70.72–82.08

CI, confidence interval; NA, not applicable

*Calculated over the population of continent patients at baseline as incidence proportion

### Evolution of pre-existing urinary incontinence

A total of 146 (59.1%) patients experienced a worsening of UI at 30 days after surgery (Table 4). At this time point, 130 (52.4%) patients reported a worsening of UI frequency and 125 (50.6%) reported an increased amount of urine loss (i.e., worsening). At 90 days, the proportion of patients with UI worsening modestly increased compared to 30 days (Table 4).

**Table 4:** Worsening of urinary incontinence at 30 and 90 days after surgery

	30 days	90 days
**UI worsening**, *n (%)*	247	228
No	101 (40.9)	85 (37.3)
Yes	146 (59.1)	143 (62.7)
**UI frequency worsening**, *n (%)*	248	229
No	118 (47.6)	99 (43.2)
Yes	130 (52.4)	130 (56.8)
**Urine amount worsening**, *n (%)*	247	228
No	122 (49.4)	97 (42.5)
Yes	125 (50.6)	131 (57.5)

UI, urinary incontinence

The scores and responses to the ICIQ-SF throughout the study are summarized in Supplementary Table S1. Overall, median (Q1;Q3) ICIQ-SF scores increased from 6.00 (1.00;13.0) points at baseline to 11.0 (5.00;13.0) points at 30 days and to 12.0 (4.00;14.0) points at 90 days AHFS. Regarding UI grades, the absolute and relative frequencies of patients with moderate, severe, and very severe UI increased at 30 days after HFS.

### Factors Associated with urinary incontinence incidence and worsening

In a multivariate log-binomial model including UTIs, AUR, constipation, prolonged catheterization, age, sex, and non-prosthetic surgery as risk factors, AUR was significantly associated with a two-fold increased risk of developing UI at this time point (*p* = 0.006) (Table 5). Age (≥ 80 years) also increased UI risk by almost two-fold, but failed to reach statistical significance.

**Table 5:** Factors associated with urinary incontinence incidence and worsening at 90 days (*n* = 84)

Predictors	Risk Ratio	Std. Error	95% CI	*p*-value	
**Incidence of Urinary Incontinence**					
Urinary tract infection	0.92	0.27	0.52–1.62	0.774	
Acute urinary retention	2.03	0.52	1.22–3.36	**0.006**	
Constipation	0.94	0.25	0.55–1.59	0.818	
Prolonged use of catheter (> 24 h)	0.74	0.15	0.50–1.10	0.141	
Age ≥80	1.85	0.60	0.97–3.50	0.060	
Sex (Male)	0.87	0.20	0.55–1.37	0.539	
Non-prosthetic surgery	0.98	0.17	0.70–1.38	0.906	
**Worsening of Urinary Incontinence**					
Urinary tract infection	1.39	0.20	1.06–1.84	**0.018**	
Prolonged use of catheter (> 24 h)	1.01	0.10	0.83–1.23	0.905	
Age ≥80	1.17	0.19	0.85–1.61	0.33	
Non-prosthetic surgery	0.99	0.10	0.81–1.21	0.934	

CI, confidence interval

Bold figures indicate statistical significance (*p* < 0.05)

Regarding factors associated with UI worsening at 90 days after HFS, UTI was significantly associated (*p* = 0.018), showing that patients with UTIs had a 39% more probability of UI worsening (Table 5).

## Discussion

This prospective cohort study including older patients undergoing HFS showed a significant incidence of UI at 30 and 90 days after HFS. Even though UI was highly prevalent at baseline, HFS resulted in an even increased prevalence and worsening of UI. Over half of patients experienced prolonged catheterization after surgery (> 24 h). Regarding related factors, AUR was identified as a risk factor for developing UI after HFS and UTIs were identified as a risk factor for UI worsening after HFS.

To our knowledge, only one previous study has prospectively examined the incidence of UI in both men and women after HFS [11], and almost all data available to date regarding the incidence of UI after HFS in the older population has been obtained from retrospective studies. These have pointed out to an increased prevalence of UI following HF, from 20 to 43% in the first study, published in 1977 [14], and from 11 to 43% in more recent studies [15, 16]. Some other prospective studies were performed exclusively in women populations and, therefore, did not reflect data from the overall population undergoing HFS [17]. Results from this prospective study including both sexes, showed increased UI prevalence by 17.34% at 30 days after surgery and 14.65% at the 90 days post-surgery follow-up.

This study focused on assessing incidence rates, showing a substantial incidence (i.e., 61.7% at 30 days) of UI after HFS. Besides increased prevalence and incidence, HFS was associated with worsening of the already existing UI. This condition was worsened in most patients with previous UI, while a subset of patients with no UI remained continent, suggesting that the former are more prone to worsen their condition than the latter. In this regard, routine procedures performed for HFS may have a higher impact on pre-existing UI. Our findings highlight the need to modify existing protocols to reduce the occurrence of UI.

To our knowledge, studies assessing UI incidence after HFS are scarce, and the prospective ones have not evaluated risk factors potentially associated with developing UI after HFS [11]. Using log-binomial models including demographic and clinical variables, we found that AUR was associated with an increased risk of UI by two-fold, and UTIs were associated with an increased risk of UI worsening. After HF, UTI poses a significant risk to patients’ recovery [18]. In addition, UTIs are a risk factor for the development of delirium, which extends the average hospital stay by 2.5 days and may increase the mortality rate [19]. The reported prevalence of UTIs after HF ranges from 12 to 61% [20] and recent studies support a correlation between UTIs and UI in older females. A recent systematic review comprising 29 studies and 518,465 women aged 55 to 106 years indicates that UTIs influence the incidence of UI (*p* < 0.001) [21]. However, to date, and despite the fact that both UTIs and UI are common problems in patients after HFS, no previous studies have found this association, although they have not made an in-depth analysis. Future research is needed to confirm the findings from this study regarding the role of UTIs in UI worsening.

In addition to UTIs, AUR was associated with a two-fold increase in the incidence of UI at 90 days after HFS. This condition is more prevalent in older individuals [22], and the literature indicates that it frequently occurs in patients with HF [23, 24]. AUR is a condition related to the patient’s hospital management, specially to traumas of the urethra caused by the use of urinary catheters [25]. Also, AUR has been linked to other medical conditions, such as UTIs and constipation [25]. The relationships between these factors are complex and bidirectional, and therefore, as the results of this study indicate, they should be carefully considered in patients undergoing HFS. Therefore, AUR and its potential causes, such as UTIs, constipation, and the inappropriate use of urinary catheters, should be managed to prevent UI in patients after HFS.

Additionally, our results pointed to age ≥ 80 years as a risk factor for the incidence of UI, although it did not reach statistical significance. It is well known that UI is highly associated with advanced age, both in women and men [26, 27]. A recent study also identified a relationship between age and UI incidence in women with HF [17]. However, data from Tuulikki et al. only identified an association with patients older than 90 years, whereas our analysis suggests an association with individuals of younger ages. Nevertheless, age is not a modifiable risk factor. The common hospital practice of using diapers on continent patients is another well-known and modifiable risk factor that, while not addressed in our study, should not be overlooked in the clinical care of elderly individuals with hip fractures [28].

The results of his study should be interpreted considering some limitations mainly associated with its setting, with patients coming from one region of Spain, potentially limiting the number of included patients. However, patients were recruited over two years, and the study sample exceeded the minimum number of patients needed to assess the primary objective (i.e., UI incidence). Nevertheless, the 248 patients recruited may have been insufficient to analyze risk factors with sufficient statistical power. Specifically, the limited number of events (patients with new-onset incontinence) precluded the inclusion of a substantial number of variables in the multivariate models to study risk factors. The researchers selected some of the most relevant variables, but others, such as patients’ pre-existing conditions or chronic medications, could not be included in the models. Additionally, the use of diapers during the hospital stay, and certain geriatric-specific variables, like the frailty syndrome, were not captured in the database, and therefore, their effects have not been considered in this study.

Although UI prevalence and incidence rates were similar to those previously reported, other study findings may not apply to other populations and settings. Regarding the primary variable, to our knowledge, this is currently the first study assessing UI after HFS with the ICIQ-SF, precluding direct comparisons with previous studies using other UI measures. Nevertheless, the available data is heterogeneous, as several published studies used non-standardized variables, precluding comparisons. Of note, the ICIQ-SF has been compared with the accepted and widely used ISI and its clinical use has been fully validated. Moreover, ICIQ-SF is a self-reported questionnaire and may have been influenced by patients’ subjectivity [29], although this circumstance can also occur in physician-performed measures [30]. In this regard, a potential information bias due to patient misreporting could not be discarded. Finally, as patients entered the study following a hip fracture, we were unable to investigate changes in the quality of life of these individuals, which would have been valuable, due to the absence of pre-fracture quality of life data.

Among the strengths of this study, to our knowledge, this is the first one, to date, reporting a follow-up period of UI after HFS, which also performs the most in-depth research on the possible causes of the incidence and worsening of this condition. Additionally, this study focused on the geriatric population, in which both UI and HFs occur in higher percentages.

Despite the impact of UI on older individuals and the high incidence of UI in HF patients, no protocols aiming to reduce the incidence of UI after HFS have been established [11]. However, some interventions have been proven effective in reducing the incidence of UI following HFS [11, 31]. Our study indicates that AUR and UTIs should be avoided. In this context, strategies to prevent UTIs, such as early patient mobilization, appropriate hydration, prevention of constipation, and avoiding catheter use should be implemented to improve patient management after HFS. Ideally, these strategies should be included in standard clinical practice to improve patient outcomes after HFS, including UI.

## Conclusions

In conclusion, this is the first prospective study to report the incidence of UI after HFS in older patients and analyze the associated risk factors. The findings of this prospective cohort study demonstrated a substantial incidence of UI among older patients after undergoing HFS, resulting in increased UI prevalence and worsening in patients with already existing UI. UTIs and AUR were identified as modifiable risk factors for developing UI in this population and should be considered in routine practice to prevent and decrease UI incidence. The results from this study underscore the need to improve current patient management protocols to decrease or eliminate the incidence of UI in older patients with HF.

### Electronic supplementary material

Below is the link to the electronic supplementary material.

Supplementary Material 1

## Data Availability

Data are available from the Consorci Sanitari Alt’Pènedes i Garraf (contact: reserca@csapg.cat) for researchers who meet the criteria for access to confidential data.
