# Supplementary material for: Incidence of urinary incontinence after hip fracture surgery and associated risk factors: a prospective study
Source: BMC Geriatr. 2024 Jan 2;24:3. doi: 10.1186/s12877-023-04597-4 (PMC10763427; doi:10.1186/s12877-023-04597-4)
Supplement: Supplementary file 1 — Supplementary Material 1 [file 12877_2023_4597_MOESM1_ESM.docx]

# Supplementary Information for the Manuscript “Incidence of Urinary Incontinence After Hip Fracture Surgery and Associated Risk Factors: A Prospective Study” Arroyo-Huidobro et al.

# Supplementary Tables

**Table S1**. Summary of ICIQ-SF responses and scores throughout study visits.

|  | **Baseline** | **30 days** | **90 days** |
| --- | --- | --- | --- |
| **Frequency, *n*** | 248 | 248 | 229 |
| About once a week or less often, *n (%)* | 17 (6.85) | 22 (8.87) | 17 (7.42) |
| 2-3 times a week, *n (%)* | 29 (11.7) | 33 (13.3) | 14 (6.11) |
| About once a day, *n (%)* | 23 (9.27) | 18 (7.26) | 11 (4.80) |
| Several times a day, *n (%)* | 52 (21.0) | 57 (23.0) | 21 (9.17) |
| All the time, *n (%)* | 25 (10.1) | 65 (26.2) | 112 (48.9) |
| Incontinence (total), *n (%)* | 146 (58.9) | 195 (78.6) | 175 (76.4) |
| Never, *n (%)* | 102 (41.1) | 53 (21.4) | 54 (23.6) |
| **Amount,** ***n*** | 248 | 247 | 228 |
| None, *n (%)* | 95 (38.3) | 53 (21.5) | 53 (23.2) |
| A small amount, *n (%)* | 74 (29.8) | 59 (23.9) | 35 (15.4) |
| A moderate amount, *n (%)* | 60 (24.2) | 63 (25.5) | 31 (13.6) |
| A large amount, *n (%)* | 19 (7.66) | 72 (29.1) | 109 (47.8) |
| **Affectation,** ***n*** | 237 | 246 | 228 |
| *Mean (SD)* | 3.5 (3.2) | 3.0 (2.5) | 2.82 (2.5) |
| **Time of UI, *n*** | 248 | 248 | 248 |
| Never, *n (%)* | 77 (31.0) | 44 (17.7) | 54 (21.8) |
| Before arriving at the bathroom, *n (%)* | 92 (37.1) | 62 (25.0) | 39 (15.7) |
| When coughing/sneezing, *n (%)* | 60 (24.2) | 54 (21.8) | 31 (12.5) |
| While sleeping, *n (%)* | 49 (19.8) | 70 (28.2) | 3 7 (14.9) |
| When performing a physical effort, *n (%)* | 35 (14.1) | 34 (13.7) | 24 (9.68) |
| After finishing urinating and dressing, *n (%)* | 10 (4.03) | 14 (5.65) | 2 (0.81) |
| No reason, *n (%)* | 32 (12.9) | 37 (14.9) | 31 (12.5) |
| Continuously, *n (%)* | 31 (12.5) | 63 (25.4) | 79 (31.9) |
| **Urination (caregiver perspective), *n*** | 238 | 244 | 227 |
| Continent, *n (%)* | 102 (42.9) | 66 (27.0) | 57 (25.1) |
| Occasionally, *n (%)* | 57 (23.9) | 46 (18.9) | 29 (12.8) |
| Incontinent, *n (%)* | 79 (33.2) | 132 (54.1) | 141 (62.1) |
| **ICIQ-SF, *n*** | 237 | 245 | 227 |
| *Median (Q1;Q3)* | 6.00 (1.00;13.0) | 11.0 (5.00;13.0) | 12.0 (4.00;14.0) |
| **Categories, n** | | | |
| Mild (1–5), *n (%)* | 106 (44.7) | 67 (27.3) | 66 (29.1) |
| Moderate (6–12), *n (%)* | 69 (29.1) | 114 (46.5) | 91 (40.1) |
| Severe (13–18), *n (%)* | 55 (23.2) | 61 (24.9) | 58 (25.6) |
| Very severe (19–21), *n (%)* | 7 (2.95) | 3 (1.22) | 12 (5.29) |
| ICIQ-SF, International Consultation on Incontinence Questionnaire - Short Form; UI, urinary incontinence. | | | |
